# Optical coherence tomography features and visual prognosis in vitreoretinal lymphoma: a structured phenotyping study

**DOI:** 10.3389/fmed.2026.1848089

**Published:** 2026-06-17

**Authors:** Xiaona Wang, Dehai Liu, Yingyu Li, Pei Zhang, Hongliang Dou, Hong Qi

**Affiliations:** Department of Ophthalmology, Peking University Third Hospital, Beijing Key Laboratory of Restoration of Damaged Ocular Nerve, Beijing, China

**Keywords:** intraocular lymphoma, intravitreal methotrexate, optical coherence tomography, retinal pigment epithelium, vitreoretinal lymphoma

## Abstract

**Purpose:**

To characterize optical coherence tomography (OCT) features of vitreoretinal lymphoma (VRL) at baseline, identify features associated with visual prognosis, and describe longitudinal OCT changes following intravitreal methotrexate (IVT-MTX) treatment.

**Design:**

Retrospective, single-center, observational study.

**Methods:**

Consecutive biopsy-proven VRL cases were included. Spectral-domain OCT (SD-OCT) features were classified into three compartments: sub-retinal pigment epithelium (sub-RPE), subretinal, and intraretinal deposits. Additional features included ellipsoid zone (EZ) disruption, EZ-RPE attenuation, preretinal deposits, and vitreous cells. Patients were stratified as isolated VRL or VRL with concurrent central nervous system involvement (VRL-CNS). Best-corrected visual acuity (BCVA) was analyzed as a continuous variable (logMAR) using generalized estimating equations (GEE) with patient-level clustering, and an exploratory LASSO model was developed to predict significant vision loss (logMAR ≥ 0.5). A subgroup receiving standardized IVT-MTX with longitudinal OCT was analyzed across treatment phases.

**Results:**

Thirty-eight eyes of 25 patients (14 female; mean age 63.5 ± 12.6 years) were included over a median follow-up of 14.2 months. Sub-RPE deposits were the most prevalent OCT feature (92.1%), followed by subretinal deposits (65.8%) and intraretinal deposits (23.7%). In the multivariable GEE model, baseline BCVA (*p* = 0.009) and EZ disruption (*p* = 0.026) were associated with worse visual outcome. In the LASSO model, EZ disruption and EZ-RPE attenuation were selected, and showed an AUC of 0.929 (0.872–0.992) for significant vision loss. Twelve eyes of 8 patients comprised the IVT-MTX longitudinal subgroup (mean 7.7 ± 0.8 injections). OCT features showed that vitreous cells resolved rapidly, sub-RPE and subretinal deposits regressed slowly, whereas EZ disruption remained unchanged.

**Conclusion:**

Sub-RPE deposits are the most prevalent OCT finding in VRL. EZ disruption and EZ-RPE attenuation are associated with worse visual prognosis. Following IVT-MTX treatment, OCT reveals differential response patterns across lesion compartments, supporting quantitative OCT assessment as a sensitive, noninvasive tool for treatment monitoring.

## Introduction

Primary central nervous system lymphoma (PCNSL) is a highly aggressive malignancy of the central nervous system (CNS) that mainly affects older adults (1). With population aging and increasing life expectancy, the burden of PCNSL-spectrum disease is expected to rise further, making the care of elderly patients an increasingly important clinical challenge (2). Vitreoretinal lymphoma (VRL) is an ocular subtype of PCNSL, with 35 to 90% of patients showing CNS involvement during the disease course (3, 4). In the United States, the annual incidence of primary VRL is approximately 0.23 per 1,000,000 and appears to be increasing, predominantly affecting individuals aged ≥60 years (5). Due to its low incidence, insidious onset, and propensity to masquerade as inflammatory or infectious uveitis, VRL frequently incurs diagnostic delays of months to years (6). This diagnostic challenge is particularly pronounced in older patients, who often have coexisting ocular conditions such as cataract and age-related macular degeneration that complicate differential diagnosis. Additionally, vitreous opacities in elderly individuals are often attributed to age-related vitreous degeneration, further delaying recognition of malignancy. The reduced tolerance for invasive vitreoretinal biopsy in elderly patients complicated by comorbidity, frailty, and reduced functional reserve also limits the feasibility of repeated diagnostic procedures (2, 7). These age-related barriers underscore the need for an accessible, noninvasive tool capable of both early detection and longitudinal treatment monitoring.

The definitive diagnosis of VRL currently relies on invasive procedures, including cytokine analysis (principally interleukin-10 [IL-10] and interleukin-6 [IL-6]), vitreous biopsy for cytopathological examination, and occasionally chorioretinal biopsy (8, 9). The diagnostic yield of vitreous cytology remains limited due to the paucity and fragility of malignant lymphocytes from small amounts of vitreous fluid (10). In recent years, spectral-domain optical coherence tomography (SD-OCT) has emerged as a critical imaging modality in the noninvasive evaluation of VRL. A recent meta-analysis summarized common OCT findings in VRL, including retinal pigment epithelium (RPE) infiltration, RPE hyperreflective material, and subretinal hyperreflective material (11).

Intravitreal methotrexate (IVT-MTX) is the most widely employed local therapy for VRL (12). However, assessment of treatment response remains difficult because VRL lacks a measurable tumor mass and often relies on observer-dependent grading of vitreous haze and subjective assessment of retinal infiltration (3). OCT enables comparative, quantitative monitoring of retinal lesion evolution and may therefore be particularly useful in older patients in whom objective and noninvasive monitoring is especially desirable (13). Zhao et al. (14) described longitudinal OCT changes in 10 patients receiving IVT-MTX, demonstrating progressive resolution of sub-RPE deposits and subretinal fluid. Jiang et al. (15) similarly reported treatment-associated OCT changes in a larger retrospective cohort.

Nevertheless, several gaps remain in the current literature. Most studies employ limited OCT phenotyping without systematic subclassification of infiltration patterns or quantitative measurement of lesion dimensions, and the prognostic significance of specific OCT features, particularly intraretinal vertical hyperreflective lesion (VHRL) (16) and ellipsoid zone (EZ) integrity (17), remains poorly defined. Therefore, the present study aims to (1) comprehensively characterize baseline OCT features in a Chinese cohort of biopsy-proven VRL using a standardized three-compartment framework with quantitative measurements, (2) explore the association of baseline OCT features with visual prognosis, and (3) describe the longitudinal OCT changes across defined IVT-MTX treatment phases.

## Methods

### Study design and participants

This retrospective observational study was conducted at Peking University Third Hospital between January 2018 and January 2026, in adherence with the tenets of the Declaration of Helsinki. The Medical Ethics Committee of the Peking University Third Hospital approved the study protocol. Informed consent was obtained from all participants. The study comprised two analyses: Part 1 included all eligible eyes for baseline OCT characterization and exploratory prognostic analysis; Part 2 included the subset of eyes that received a standardized, complete IVT-MTX treatment protocol (encompassing induction, consolidation, and maintenance phases) and had interpretable SD-OCT images at each treatment time point, constituting the longitudinal subgroup.

Patients were included if they met: (1) cytological or histopathological confirmation of VRL from vitreous or retinal biopsy, or histopathologically confirmed CNS lymphoma with characteristic intraocular findings consistent with VRL, following exclusion of uveitis (18); (2) at least 3 months of follow-up with available data on best-corrected visual acuity (BCVA). Based on CNS involvement status, eyes were classified as isolated VRL or VRL with concurrent CNS involvement (VRL-CNS). Exclusion criteria included co-existing macular pathology (e.g., age-related macular degeneration, diabetic macular edema), or poor-quality OCT images precluding reliable interpretation. For the baseline cross-sectional analysis, features not assessable due to media opacity were coded as ‘not gradable’ and excluded from the feature-specific prevalence calculation. For the longitudinal subgroup analysis, patients with missing OCT data at any required time point were excluded.

### Treatment protocol

All included patients received local ocular therapy. IVT-MTX (400 μg/0.1 mL) was administered following a modified protocol, comprising an induction phase of once-weekly injections for 1 month, a consolidation phase of one injection every 2 weeks for 1 month, and a maintenance phase of monthly injections with subsequent tapering based on clinical and OCT assessment. Detailed protocols were described and validated in the prior publication (19). When CNS involvement was present, the systemic therapeutic regimen was codetermined by the patient and the oncology team following multidisciplinary assessment.

### OCT image acquisition and analysis

**Figure 1:**
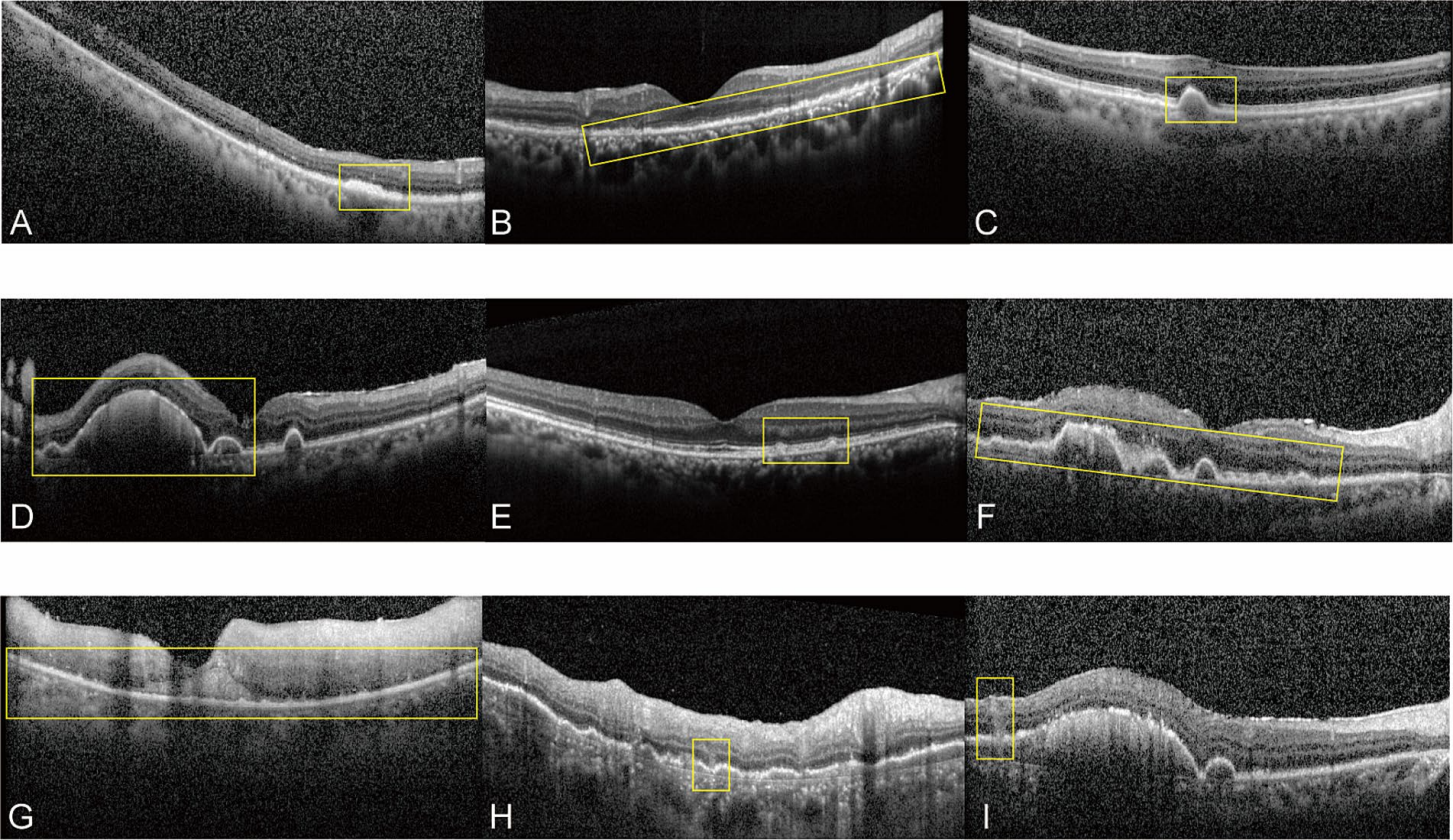
Representative spectral-domain optical coherence tomography features of vitreoretinal lymphoma classified by anatomical compartment. Sub-RPE deposits: **(A)** focal thickened RPE—localized hyperreflective elevation of the RPE band; **(B)** diffuse thickened RPE—broad, plaque-like RPE thickening extending over multiple B-scans; **(C)** shallow PED—maximal height <250 μm; **(D)** large PED—maximal height ≥250 μm. Subretinal deposits: **(E)** focal round—localized hyperreflective nodules; **(F)** band-like—confluent hyperreflective material distributed along the RPE; **(G)** band-like with vitelliform lesions—confluent hyperreflective material with vitelliform morphology. Intraretinal deposits: **(H)** incomplete VHRL—a vertically oriented hyperreflective band partially traversing the neuroretina; **(I)** complete VHRL—a vertically oriented hyperreflective band spanning all layers of the neuroretina between the retinal nerve fiber layer and the RPE. RPE, retinal pigment epithelium; PED, pigment epithelial detachment; VHRL, vertical hyperreflective retinal lesion.

High-resolution macular images centered on the fovea were acquired using SD-OCT (Spectralis HRA + OCT; Heidelberg Engineering, Heidelberg, Germany), obtained using a 30° volume scan pattern with an 8.8 × 8.8 mm scanning area positioned at the center of the fovea. Two trained graders, masked to clinical information and CNS involvement status, independently evaluated all OCT images, with disagreements resolved by consensus with a senior reader. OCT features were recorded if present on any B-scan within the volume. For quantitative analyses, lesion dimensions were measured manually using the built-in caliper tool in Heidelberg Eye Explorer software. Intergrader reliability was assessed using Cohen’s kappa statistics. OCT features were classified into three compartments following the framework of Pichi et al. (4): sub-RPE deposits (subtyped as thickened RPE [focal or diffuse], shallow pigment epithelial detachment [PED] with a maximal height of less than 250 μm, or large PED with a maximal height of more than 250 μm), subretinal deposits (subtyped as focal round, band-like, or band-like with vitelliform lesions), and intraretinal deposits (subtyped as incomplete or complete VHRL). Figure 1 presents representative imaging examples. Detailed definitions of these OCT features have been described previously (4, 18). In the longitudinal subgroup, sub-RPE and subretinal deposits were quantified by maximum height (measured perpendicular to the RPE or Bruch’s membrane plane) and width (measured along the horizontal extent) of the single largest deposit on OCT scans (20). In eyes with confluent deposits, the overall maximum height and horizontal extent were measured. Additional features assessed included EZ disruption, EZ-RPE attenuation, central foveal thickness (CFT), preretinal deposits, and vitreous cells. EZ disruption was defined as full-thickness discontinuity of the EZ band on SD-OCT. EZ-RPE attenuation was defined as an EZ-to-RPE thickness <20 μm (21). CFT was automatically generated by the OCT device within the central 1-mm ETDRS subfield. Vitreous haze was graded on a scale from 0.5 to 4 per the Standardization of Uveitis Nomenclature (SUN) Working Group criteria (22). For the longitudinal subgroup, serial SD-OCT imaging was performed at five predefined time points: pre-treatment (baseline), post-induction, post-consolidation, 1 month post-maintenance, and last follow-up (14). BCVA at each longitudinal visit was recorded in logMAR units; count fingers, hand motion, light perception, and no light perception were assigned logMAR values of 1.7, 2.0, 2.3, and 3.0, respectively.

### Statistical analysis

Continuous variables were tested for normality using the Shapiro–Wilk test. Normally distributed variables are presented as mean ± standard deviation (SD); non-normally distributed variables as median (interquartile range [IQR]). Categorical variables were expressed as *n* (%). Subgroup comparisons between isolated VRL and VRL-CNS used the Fisher exact test for categorical variables and the Mann–Whitney *U* test for continuous variables. To account for intra-patient correlation in bilateral cases, the exploratory prognostic analysis was performed using generalized estimating equations (GEE) with an independent working correlation structure, with patient as the clustering unit. Each baseline OCT feature was evaluated in a separate age- and sex-adjusted GEE model with last follow-up BCVA (logMAR) as the dependent variable; *β* coefficients, 95% confidence intervals (CI), and *p* values are reported. To control for multiple comparisons, the Benjamini–Hochberg false discovery rate (FDR) correction was applied. Variables reaching *p* < 0.05 in the individual screening models were entered into a parsimonious multivariable GEE model. As an exploratory analysis, LASSO-penalized logistic regression (L1 regularization) was used to predict significant vision loss (defined as last BCVA ≥ 0.5 logMAR) from baseline clinical and OCT features. Model performance was assessed using an optimism-corrected area under the curve (AUC) with 95% bootstrap percentile CI (1,000 replications). Sensitivity and specificity were reported at the optimal Youden index threshold. For the longitudinal subgroup, we supplemented the Wilcoxon signed-rank tests with a linear mixed-effects model incorporating patient as a random intercept and treatment phase as a fixed effect. Statistical significance was set at two-sided *p* < 0.05. All analyses were performed using R software (version 4.4.1).

## Results

### Patient characteristics

**Figure 2:**
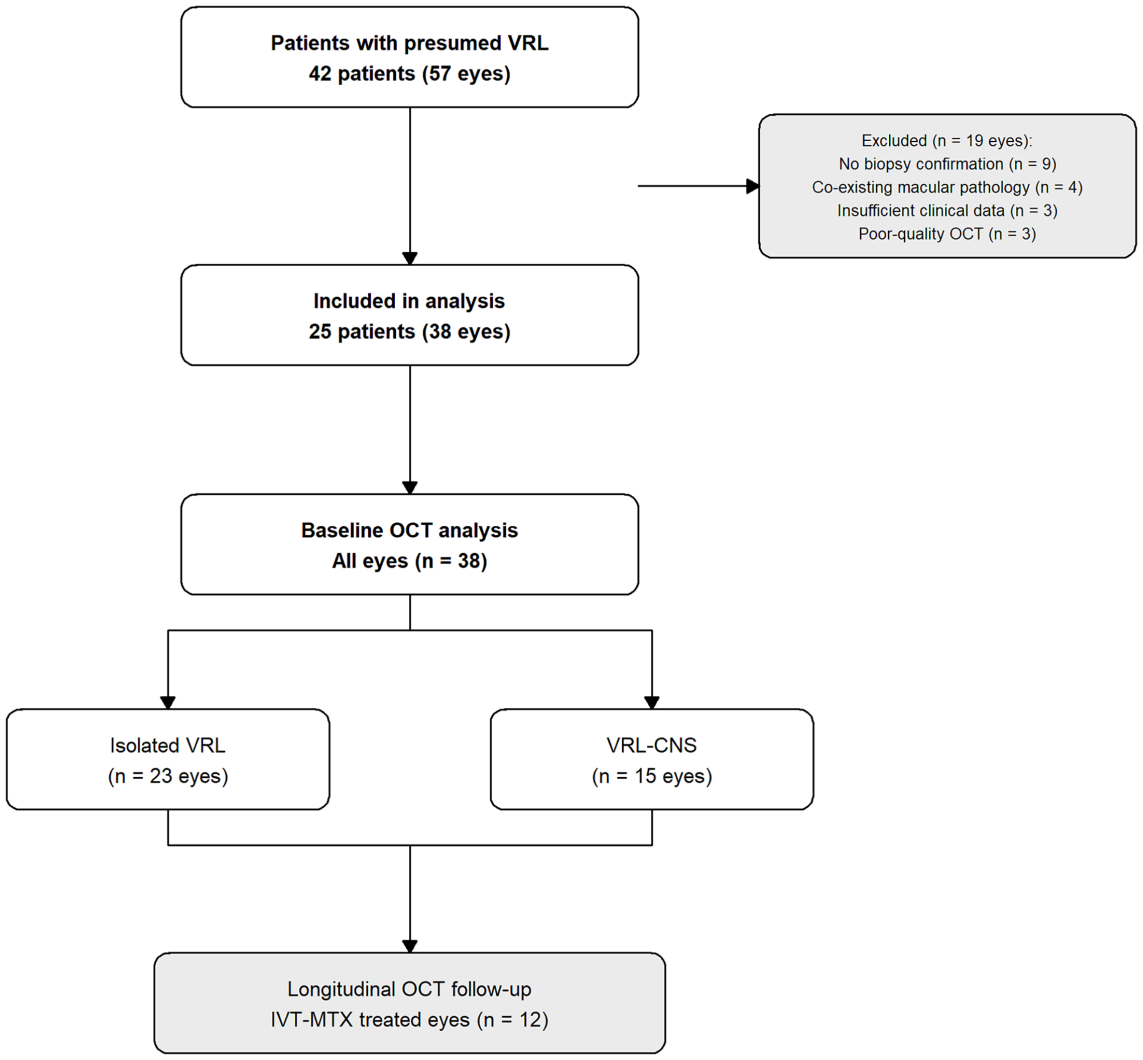
Patient selection flowchart. VRL, vitreoretinal lymphoma; VRL-CNS, vitreoretinal lymphoma with concurrent central nervous system involvement; OCT, optical coherence tomography; IVT-MTX, intravitreal methotrexate.

Figure 2 illustrates the patient selection process. From 42 patients (57 eyes) with presumed VRL, 38 eyes of 25 patients met the inclusion criteria after excluding 19 eyes for the following reasons: absence of biopsy confirmation (*n* = 9), co-existing macular pathology (*n* = 4), insufficient clinical data (*n* = 3), or poor-quality OCT (*n* = 3). VRL was confirmed in all 38 eyes: 21 eyes by vitreous biopsy alone, 2 by combined vitreous and chorioretinal biopsy, 9 by combined brain and vitreous biopsy, and 6 by brain biopsy with characteristic intraocular findings.

Table 1 presents the baseline demographic and clinical characteristics of the final cohort. The mean age was 63.5 ± 12.6 years (range 41–89 years), 14 patients (56.0%) were female, and 13 (52.0%) had bilateral involvement. Twenty-three eyes (60.5%) were classified as isolated VRL and 15 (39.5%) as VRL-CNS. Twenty-four of 38 eyes (63.2%) experienced delayed or incorrect initial diagnosis, including 9 (23.7%) misdiagnosed as uveitis and 15 (39.5%) with delayed diagnosis after prolonged evaluation. All 25 patients (38 eyes) underwent IVT-MTX, with a mean of 7 ± 1 injections (range 5–9). All 15 patients in the VRL-CNS subgroup received systemic chemotherapy. Thirty-two eyes (84.2%) underwent diagnostic pars plana vitrectomy.

**Table 1:** Baseline demographic and clinical characteristics.

Characteristics	All (*n* = 38 eyes)	Isolated VRL (*n* = 23 eyes)	VRL-CNS (*n* = 15 eyes)	*p*-value
Age, years, mean ± SD	63.5 ± 12.6	67.8 ± 12.3	56.9 ± 10.1	0.003
Female, *n* (%)	21 (55.3)	14 (60.9)	7 (46.7)	0.509
Bilateral involvement, *n* (%)	26 (68.4)	16 (69.6)	10 (66.7)	1.000
Baseline BCVA, logMAR, median (IQR)	0.4 (0.22–0.82)	0.4 (0.22–1)	0.4 (0.1–0.61)	0.191
Follow-up BCVA, logMAR, median (IQR)	0.22 (0.02–0.79)	0.22 (0.11–1.3)	0.22 (0.05–0.37)	0.136
BCVA change from baseline, *n* (%)				1.000
Improved ≥0.1 logMAR, *n* (%)	19 (50.0)	11 (47.8)	8 (53.3)	
Stable, *n* (%)	14 (36.8)	9 (39.1)	5 (33.3)	
Worsened ≥0.1 logMAR, *n* (%)	5 (13.2)	3 (13)	2 (13.3)	
Delayed/incorrect initial diagnosis, *n* (%)	24 (63.2)	16 (69.6)	8 (53.3)	0.492
Misdiagnosed as uveitis, *n* (%)	9 (23.7)	6 (26.1)	3 (20.0)	
Delayed diagnosis, *n* (%)	15 (39.5)	10 (43.5)	5 (33.3)	
Systemic chemotherapy, *n* (%)	18 (47.4)	3 (13)	15 (100.0)	< 0.001
Median follow-up, months (IQR)	14.2 (6.9–27.5)	13.4 (8.2–35.9)	15.0 (7.4–19.1)	0.310

BCVA, best-corrected visual acuity; logMAR, logarithm of the minimum angle of resolution; IQR, interquartile range; SD, standard deviation; VRL, vitreoretinal lymphoma; VRL-CNS, VRL with concurrent central nervous system involvement.

### Baseline OCT features

Table 2 displays the baseline OCT features of the entire cohort and by CNS involvement subgroup. Sub-RPE deposits were the most prevalent finding (35/38 eyes, 92.1%), comprising four morphological subtypes: thickened RPE (focal) (16/35, 45.7%), thickened RPE (diffuse) (9/35, 25.7%), shallow PED (5/35, 14.3%), and large PED (5/35, 14.3%). Subretinal deposits were present in 25/38 eyes (65.8%), with three morphological subtypes: focal round (11/25, 44.0%), band-like (10/25, 40.0%), and band-like with vitelliform lesions (4/25, 16.0%). Intraretinal deposits were observed in 9/38 eyes (23.7%) and were subclassified as incomplete VHRL (4/9, 44.4%) and complete VHRL (5/9, 55.6%). No significant differences in OCT features were observed between the two subgroups (all *p* > 0.05). Analysis of infiltration patterns revealed that sub-RPE combined with subretinal deposits was the most common pattern (16/38, 42.1%), followed by sub-RPE deposits alone (10/38, 26.3%), and sub-RPE with subretinal and intraretinal deposits (9/38, 23.7%). All 9 eyes with intraretinal deposits had concurrent sub-RPE and subretinal involvement.

**Table 2:** Baseline optical coherence tomography features.

OCT feature	All (*n* = 38)	Isolated VRL (*n* = 23)	VRL-CNS (*n* = 15)	*p-*value
Sub-RPE deposits	35 (92.1)	21 (91.3)	14 (93.3)	1.000
Thickened RPE (focal)	16 (45.7)			
Thickened RPE (diffuse)	9 (25.7)			
Shallow PED	5 (14.3)			
Large PED	5 (14.3)			
Subretinal deposits	25 (65.8)	18 (78.3)	7 (46.7)	0.079
Focal round	11 (44.0)			
Band-like	10 (40.0)			
Band-like with vitelliform lesions	4 (16.0)			
Intraretinal deposits	9 (23.7)	6 (26.1)	3 (20.0)	1.000
Incomplete VHRL	4 (44.4)			
Complete VHRL	5 (55.6)			
Infiltration patterns				
Sub-RPE + subretinal	16 (42.1)	12 (52.2)	4 (26.7)	
Sub-RPE only	10 (26.3)	3 (13.0)	7 (46.7)	
Sub-RPE + subretinal + intraretinal	9 (23.7)	6 (26.1)	3 (20.0)	
No deposits	3 (7.9)	2 (8.7)	1 (6.7)	
Subretinal only	0 (0)	0 (0)	0 (0)	
Other features				
EZ disruption	15 (39.5)	11 (47.8)	4 (26.7)	0.310
EZ-RPE attenuation	9 (23.7)	7 (30.4)	2 (13.3)	0.273
Preretinal deposits	19 (50.0)	13 (56.5)	6 (40.0)	0.508
Vitreous cells	18 (47.4)	12 (52.2)	6 (40.0)	0.522
Vitreous haze, median (IQR)	2 (1–2)	2 (1–2)	2 (1–2)	0.385
CFT, μm, median (IQR)	256 (242–282)	252 (245–279)	259 (241–289)	0.560

CFT, central foveal thickness; EZ, ellipsoid zone; IQR, interquartile range; PED, pigment epithelial detachment; RPE, retinal pigment epithelium; VHRL, vertical hyperreflective lesion.

EZ disruption was found in 15/38 (39.5%), and EZ-RPE attenuation was present in 9/38 eyes (23.7%). Preretinal deposits were found in 19/38 eyes (50.0%), and vitreous cells in 18/38 (47.4%). Median vitreous haze was grade 2.0 (1.0–2.0). Inter-rater agreement was excellent for the three-compartment infiltration classification, and good to excellent for specific subtype classifications and other OCT features (all *κ* > 0.70; Supplementary Table 1).

### Follow-up and visual prognosis

The median follow-up was 14.2 months (6.9–27.5). The median baseline BCVA was 0.40 logMAR (0.22–0.82). At last follow-up, median BCVA was 0.22 logMAR (0.02–0.79), with significant overall improvement from baseline (Wilcoxon signed-rank test, *p* < 0.001). Of 38 eyes with paired BCVA data, 19 (50.0%) improved by ≥0.1 logMAR, 14 (36.8%) remained stable, and 5 (13.2%) worsened. No serious injection-related complications (endophthalmitis, retinal detachment) were documented; corneal epitheliopathy was observed in 5 eyes and transient intraocular pressure elevation in 3 eyes. No intraocular relapse was documented during the follow-up period.

Table 3 presents the results of the exploratory prognostic analysis with BCVA at last follow-up. In the individual GEE models, baseline BCVA (*β* = 0.868, 95% CI 0.637–1.100; *p* < 0.001, *Q* < 0.001), EZ-RPE signal attenuation (*β* = 1.368, 95% CI 0.784–1.953; *p* < 0.001, *Q* < 0.001), EZ disruption (*β* = 1.144, 95% CI 0.675–1.614; *p* < 0.001, *Q* < 0.001), intraretinal deposits (*β* = 0.930, 95% CI 0.208–1.651; *p* = 0.016, *Q* = 0.029), and subretinal deposits (*β* = 0.788, 95% CI 0.315–1.261; *p* = 0.003, *Q* = 0.007) were significantly associated with worse BCVA at last follow-up. In the multivariable model, baseline BCVA (*β* = 0.606, 95% CI 0.211–1.001; *p* = 0.009) and EZ disruption (*β* = 0.497, 95% CI 0.073–0.922; *p* = 0.026) remained independently significant. These results were robust in a sensitivity analysis restricted to eyes with direct intraocular biopsy confirmation (Supplementary Table 2).

**Table 3:** Generalized estimating equation (GEE) prognostic analysis of BCVA at last follow-up.

Feature	Individual analysis				Multivariable analysis		
	*β*	95% CI	*p-*value	*q-*value (FDR)	*β*	95% CI	*p-*value
Baseline BCVA (logMAR)	0.868	0.637 to 1.100	<0.001*	<0.001^†^	0.606	0.211 to 1.001	0.009*
EZ-RPE attenuation^a^	1.368	0.784 to 1.953	<0.001*	<0.001^†^			
EZ disruption	1.144	0.675 to 1.614	<0.001*	<0.001^†^	0.497	0.073 to 0.922	0.026*
Intraretinal deposits	0.930	0.208 to 1.651	0.016*	0.029^†^	0.046	−0.468 to 0.56	0.845
Sub-RPE deposits	0.572	−0.107 to 1.250	0.073	0.094			
Subretinal deposits	0.788	0.315 to 1.261	0.003*	0.007^†^	0.128	−0.140 to 0.397	0.315
Vitreous cells	0.326	−0.255 to 0.908	0.256	0.287			
Preretinal deposits	−0.081	−0.637 to 0.475	0.764	0.764			
CNS involvement	−0.519	−1.073 to 0.036	0.064	0.094			

a EZ-RPE attenuation was excluded from the multivariable model owing to its conceptual and statistical overlap with EZ disruption (Phi coefficient = 0.690; VIF = 2.32).

^†^*Q* < 0.05 (Benjamini–Hochberg false discovery rate correction).

*p < 0.05.

FDR, false discovery rate; BCVA, best-corrected visual acuity; logMAR, logarithm of the minimum angle of resolution; CI, confidence interval; CNS, central nervous system; EZ, ellipsoid zone; RPE, retinal pigment epithelium.

Table 4 presents the exploratory LASSO prognostic model for significant vision loss (last BCVA ≥ 0.5 logMAR). LASSO selected 2 features: EZ disruption and EZ-RPE attenuation. The model achieved an optimism-corrected AUC of 0.929 (95% CI, 0.872–0.992), with sensitivity of 92.9% and specificity of 91.7%.

**Table 4:** Exploratory LASSO prognostic model for significant vision loss (last BCVA ≥ 0.5 logMAR).

Feature	LASSO coefficient	Selected (yes/no)*
Age	0	No
Baseline BCVA (logMAR)	0	No
EZ-RPE attenuation	1.991	Yes
EZ disruption	0.504	Yes
Intraretinal deposits	0	No
Sub-RPE deposits	0	No
Subretinal deposits	0	No
Vitreous cells	0	No
Preretinal deposits	0	No

*Feature selected by LASSO (non-zero coefficient at lambda 1s). Outcome: significant vision loss (last BCVA ≥ 0.5 logMAR); *N* = 38 eyes; event rate = 14/38 (36.8%).

### Longitudinal OCT changes following IVT-MTX

Twelve eyes of 8 patients (7 female; mean age 64.3 years) who completed the standardized IVT-MTX protocol and had available longitudinal OCT imaging comprised the longitudinal subgroup. The mean number of injections was 7.7 ± 0.8 (range 7–9). Six eyes (50.0%) had concurrent CNS involvement.

**Figure 3:**
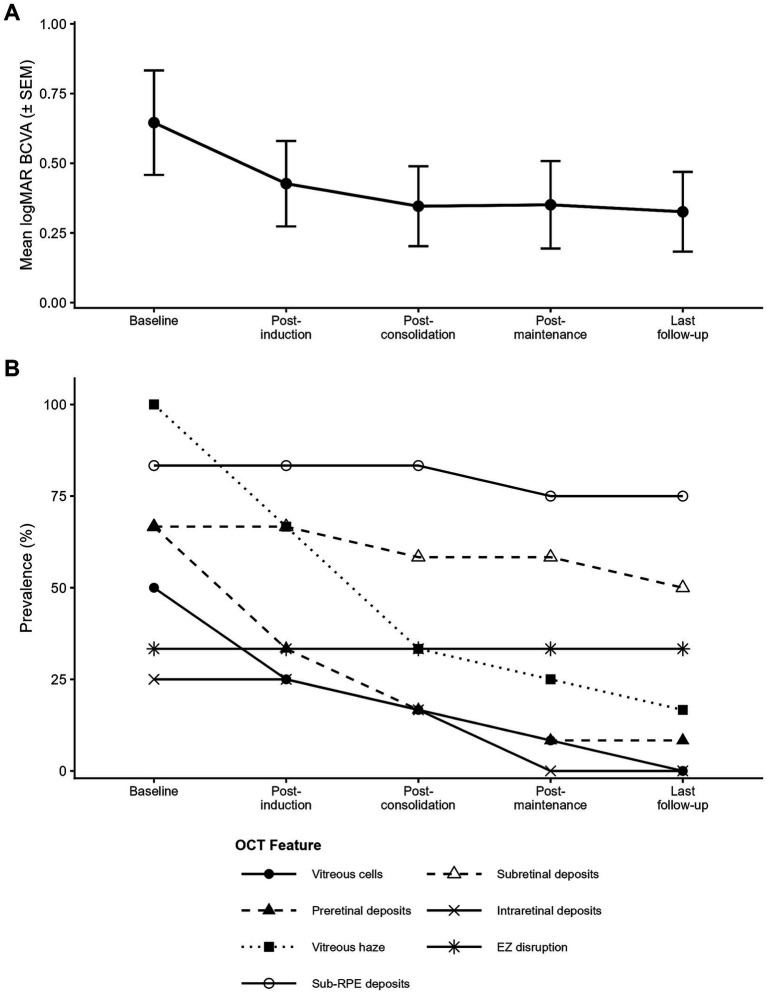
Visual acuity trajectory and optical coherence tomography (OCT) feature prevalence following intravitreal methotrexate (IVT-MTX) treatment (*n* = 12 eyes). **(A)** Mean logarithm of the minimum angle of resolution (logMAR) best-corrected visual acuity (BCVA) across five treatment phases (baseline, post-induction, post-consolidation, 1 month post-maintenance, and last follow-up). **(B)** Multi-tier treatment response model showing the prevalence (%) of seven OCT features across the same treatment phases. RPE, retinal pigment epithelium; EZ, ellipsoid zone.

**Figure 4:**
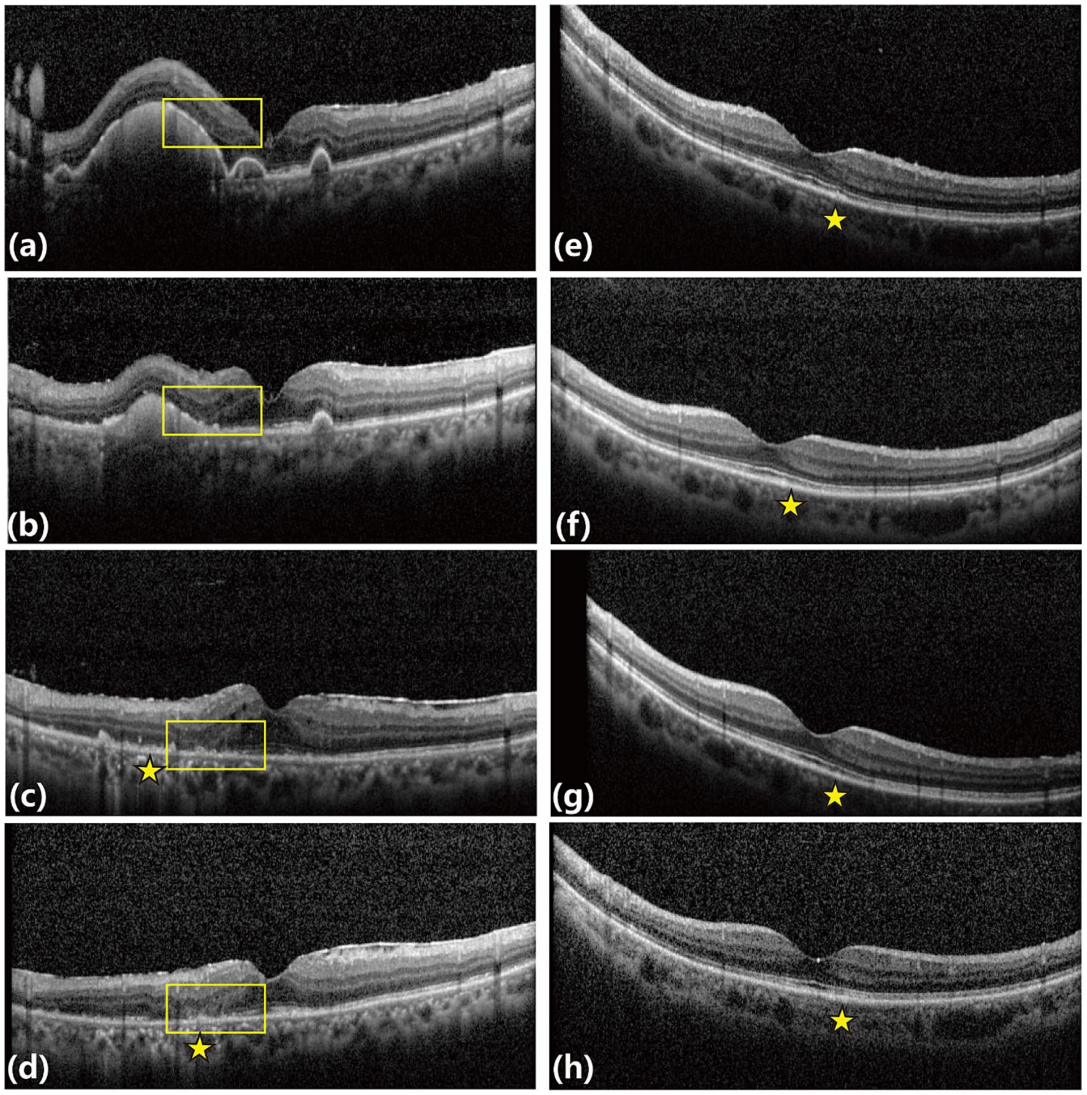
Longitudinal spectral-domain optical coherence tomography (SD-OCT) documentation of two representative cases following intravitreal methotrexate (IVT-MTX) treatment. Case 1 [**(a–d)**, right eye]: **(a)** Baseline OCT demonstrating a large pigment epithelial detachment (PED) with subretinal hyperreflective infiltration and ellipsoid zone (EZ) disruption (yellow rectangle). **(b)** Post-induction: marked reduction in PED height and decrease in subretinal hyperreflective material, while EZ disruption persists. **(c)** Post-consolidation: complete resolution of PED, replaced by residual thickened and irregular retinal pigment epithelium (RPE) (yellow asterisk); subretinal deposits further regressed. **(d)** Post-maintenance: further attenuation of RPE irregularity, whereas the EZ disruption remained. Case 2 [**(e–h)**, left eye]: **(e)** Baseline OCT revealing subfoveal thickened RPE with mild hyperreflective sub-RPE band without significant overlying retinal disruption. **(f–h)** Post-induction, post-consolidation, and post-maintenance showing gradual reduction in thickness and lateral extent of the sub-RPE deposit (yellow asterisk), with residual RPE thickening persisting at last follow-up.

Figure 3 presents the BCVA trajectory and OCT feature prevalence across treatment phases. Median BCVA improved from 0.22 logMAR at baseline to 0.10 at last follow-up (*p* = 0.006), with 9 of 12 eyes (75.0%) improving by ≥0.1 logMAR. OCT features showed a tiered response pattern. Vitreous cells resolved rapidly from 50 to 0%, and preretinal deposits decreased from 67 to 8%. Sub-RPE deposits persisted in most eyes (83 to 75%) but showed significant reductions in median height (68 to 32 μm; *p* = 0.004) and width (355 to 261 μm; *p* = 0.001), and subretinal deposits decreased in prevalence from 67 to 50% with median width decreasing from 588 to 136 μm (*p* = 0.031) (Supplementary Figure 1). Intraretinal deposits resolved completely from 25 to 0%. EZ disruption remained unchanged throughout follow-up. Figure 4 presents longitudinal OCT documentation of two representative cases.

## Discussion

This study provides a structured OCT phenotyping of biopsy-proven VRL in a Chinese cohort, and an exploratory analysis of visual prognosis and longitudinal treatment response to IVT-MTX. Sub-RPE deposits were the most prevalent OCT finding, with thickened RPE as the predominant subtype. Baseline EZ disruption and EZ-RPE attenuation were associated with worse BCVA at final follow-up. Longitudinally, OCT features showed a tiered response to IVT-MTX: vitreous and preretinal abnormalities resolved rapidly, sub-RPE and subretinal deposits regressed slowly with a measurable reduction in volume, whereas structural EZ damage persisted without significant change.

The diagnosis of VRL remains a considerable clinical challenge, particularly in the elderly population. In our cohort (mean age 63.5 years), 63.2% of patients experienced delayed or incorrect initial diagnosis, predominantly misdiagnosed as uveitis, reflecting the well-recognized propensity of VRL to masquerade as nonspecific intraocular inflammation (23). This diagnostic delay is disproportionately impactful in older patients. In PCNSL, treatment initiation is more frequently delayed in elderly patients, potentially contributing to poorer outcomes (24, 25). In parallel, Tang et al. (26) reported that 32% of patients in an international multicenter VRL cohort with median age at diagnosis of 65 years experienced a diagnostic delay of more than 12 months, highlighting the particular need for accessible early detection strategies in older adults that remain feasible even in non-tertiary settings. Vitreoretinal biopsy with cytopathological analysis remains the gold standard; however, the diagnostic yield is limited by the sparse and fragile nature of malignant lymphocytes, with false-negative rates of 30–45% (27). IL-10 and IL-6 assessment in aqueous humor provides a relatively rapid screening tool, with reported sensitivity of 88% and specificity of 85% at a threshold of IL-10/IL-6 ratio greater than 1 (28). Recent literature suggest that younger patients are increasingly represented (29), likely reflecting earlier utilization of vitreous biopsy and interleukin testing. Nevertheless, these diagnostic modalities require invasive intraocular sampling, which may be poorly tolerated by elderly patients, especially in clinical scenarios requiring early screening rather than confirmatory diagnosis. OCT, as a noninvasive, rapid, and highly reproducible imaging modality, holds promise by capturing subtle retinal architectural changes and enabling quantitative feature analysis of VRL (13). Recent landmark studies have advanced the OCT characterization of VRL. Barry et al. (30) first reported that hyperreflective subretinal infiltrates were highly suggestive of the diagnosis of VRL. Deák et al. (16) found that intraretinal VHRL was a relatively distinctive OCT feature, potentially reflecting microinfiltrates of tumor cells traversing the retinal layers. Pichi et al. (4) subsequently established a three-compartment infiltration framework classified as sub-RPE, subretinal, and intraretinal.

As summarized in Table 5, our three-compartment OCT characterization was broadly consistent with prior VRL series (4, 11, 14, 15, 18, 30–36), while showing several notable differences. Sub-RPE deposits remained the dominant finding in our cohort (92.1%), comparable to recent large cohort studies (4, 18). Subretinal deposits were also relatively frequent (65.8%). By contrast, intraretinal deposits were less common overall (23.7%) than in several recent reports (31, 33). In addition, preretinal deposits were identified in half of eyes which was more prevalent than previously appreciated (34). These between-study differences likely reflect variation in cohort composition, ethnicity, and disease stage at presentation. Although sub-RPE deposits are the most common OCT finding in patients with VRL, they are not specific. Previous studies have suggested that intraretinal VHRLs may represent a relatively more distinctive OCT feature, potentially reflecting the transretinal migration of tumor cells (16). Drusenoid pigment epithelial detachments and reticular pseudodrusen in age-related macular degeneration may produce sub-RPE hyperreflective material that morphologically overlaps with VRL-associated deposits (37, 38). In addition, inflammatory chorioretinopathies, including serpiginous choroiditis, multifocal choroiditis, sarcoid chorioretinitis, and tuberculous chorioretinitis, may also present with sub-RPE infiltrates (39, 40). Therefore, OCT findings in suspected VRL should always be interpreted in conjunction with the clinical context, including patient age, bilaterality, and examinations such as CNS imaging and IL-10/IL-6 cytokine profiling. Within this framework, OCT should be regarded as a non-invasive screening tool for patients at high risk of VRL, rather than as an independent diagnostic modality.

**Table 5:** Systematic comparison of baseline OCT feature prevalence across published VRL cohorts.

Study (author, year)	Sub-RPE (%)	Subretinal (%)	Intraretinal (%)	PED (%)	EZ disruption (%)	Vitreous cells (%)	Preretinal deposits (%)
Present study	92.1	65.8	23.7	26.3	39.5	47.4	50
The International Vitreoretinal B-cell Lymphoma Registry Investigator Group (2026) (32)	41.6	34.7	9.6	NR	NR	NR	NR
Salari (2025) (11)*	NR	39.7	19.7	15.3	39.3	79.7	NR
Chauhan (2025) (31)	NR	50	33.5	NR	NR	NR	NR
El Zein (2024) (33)	53.7	38.9	34	NR	NR	48.4	31.6
Guan (2023) (18)	80	62.2	53.3	26.7	NR	93.3	44.4
Pichi (2022) (4)	90.7	43.4	6.6	41.8	NR	NR	NR
Jiang (2022) (15)	59.5	40.5	39.6	25.2	52.3	94.6	NR
Giuffrè (2021) (35)	69.5	32.2	20.3	NR	50.8	100	NR
Yang (2021) (34)	63.6	36.4	14.5	49.1	NR	65.5	12.7
Zhao (2020) (14)	NR	16.7	44.4	50	NR	100	NR
Barry (2018) (30)	25	53.1	18.8	NR	NR	15.6	NR
Keino (2016) (36)	28.6	NR	NR	NR	38.1	80.9	NR

Values represent percentage of eyes with the specified feature at baseline. *Data derived from a meta-analysis. NR, not reported; VRL, vitreoretinal lymphoma; RPE, retinal pigment epithelium; PED, pigment epithelial detachment; EZ, ellipsoid zone.

The distribution of infiltration patterns in our cohort provides insight into the pathophysiology of VRL. Sub-RPE deposits were the dominant type, present in isolation or combined with other deposit types in 92.1% of eyes, while isolated intraretinal infiltration was never observed. This pattern is consistent with the hypothesis proposed by Pichi et al. (4) that lymphoma cells may originate from the choroidal circulation and migrate across Bruch’s membrane and the RPE, subsequently extending into the subretinal space and eventually the retina and vitreous cavity. The human tissue study also supported that B-cell chemokines secreted by RPE cells may attract malignant lymphocytes from the choroidal circulation (41). However, Chen et al. (42, 43) described perivascular flower-bud-like lesions on enface OCT angiography in VRL patients, demonstrating perivascular infiltration without accompanying RPE disruption and suggesting an alternative mechanism of lymphoma deposition via retinal vascular microinfiltration. These findings highlight the pathophysiological complexity and potential heterogeneity of VRL infiltration patterns.

Our exploratory analysis identified EZ disruption and EZ-RPE attenuation as the OCT features associated with worse visual prognosis. The EZ corresponds to the mitochondria-rich portion of the photoreceptor inner segments, and EZ disruption and EZ-RPE attenuation reflect structural discontinuity of the photoreceptor band and thinning of the outer retinal compartment between the EZ and RPE, respectively (17). Evidence suggests that EZ integrity may serve as a biomarker of photoreceptor health and predict retinal disease progression (44, 45). Using GEE analysis and LASSO prognostic model, we demonstrated for the first time that baseline EZ disruption and EZ-RPE attenuation are significantly associated with poorer visual outcome in VRL, with the combined model showing robust predictive performance. In our cohort, the prevalence of EZ disruption and EZ-RPE attenuation was 39.5 and 23.7%, respectively, suggesting that lymphoma-related photoreceptor damage is not uncommon yet may represent a relatively later manifestation compared to sub-RPE deposits which is the most prevalent baseline finding (92.1%). Consistent with Chauhan et al. (31), sub-RPE deposits were not significantly associated with visual outcome at last follow-up. Notably, prior studies have generally not distinguished intraretinal, subretinal, and sub-RPE deposits as separate prognostic variables (46), and our analysis provides a more granular dissection. In the longitudinal subgroup, EZ disruption remained unchanged during treatment. These findings suggest that outer retinal structural damage in VRL may represent relatively irreversible photoreceptor injury; however, the modest follow-up duration may have been insufficient to capture photoreceptor recovery, which has been reported over longer time in previous literature (36).

The assessment of treatment response in VRL remains a major challenge. Current international standardized guidelines for PCNSL ocular response define complete response as the absence of vitreous cells with resolution of retinal infiltration (47). However, vitreous cell quantification is inherently subjective, and grading systems vary in their precision (3). The application of OCT holds promise as a treatment response metric. In our longitudinal cohort, the rapidly resolving vitreous features including vitreous cells, preretinal deposits, vitreous haze, were most responsive to IVT-MTX, consistent with previous findings (14, 15). The slowly resolving tier including sub-RPE and subretinal deposits was clinically relevant. Although sub-RPE deposits persisted in most of eyes at last follow-up, both height and width decreased significantly. Subretinal deposits likewise showed a significant reduction in median width. These findings suggest that quantitative measurement in the volume of deposits may provide a sensitive marker of treatment response.

The major strength of this study lies in our systematic subclassification of VRL infiltration, and the inclusion of EZ integrity as a potentially prognostic marker extend the existing OCT phenotyping framework. However, several limitations should be acknowledged. First, the longitudinal subgroup was relatively small; therefore, these results should be considered preliminary. Second, the single-center retrospective design may introduce potential selection biases. Third, although inter-eye non-independence in bilateral cases was addressed using GEE, residual correlation may remain. Fourth, although all B-scans within the Spectralis 30° volume scan were reviewed, the analysis remains centered on the posterior pole and may not capture pathology in the retinal periphery. Finally, the prognostic analyses should be interpreted as exploratory and hypothesis-generating due to modest sample size.

In conclusion, sub-RPE deposits are the most prevalent OCT finding in VRL, with the thickened RPE pattern being the dominant subtype. EZ disruption and EZ-RPE attenuation are associated with worse visual prognosis. Quantitative deposit measurement in OCT may serve as a potentially sensitive treatment response metric. These findings support the integration of quantitative OCT assessment into the diagnostic evaluation and longitudinal monitoring of VRL.

## Data Availability

The raw data supporting the conclusions of this article will be made available by the authors, without undue reservation.
