# Supplementary material for: Optical coherence tomography features and visual prognosis in vitreoretinal lymphoma: a structured phenotyping study
Source: Front Med (Lausanne). 2026 Jun 17;13:1848089. doi: 10.3389/fmed.2026.1848089 (PMC13318726; doi:10.3389/fmed.2026.1848089)

**Supplementary material**

**Supplementary Table 1**. Inter-rater agreement for specific OCT features.

**Supplementary Table 2**. Sensitivity analysis of visual prognostic analysis restricted to eyes with direct intraocular biopsy confirmation.

**Supplementary Figure 1**. OCT features of sub-RPE and subretinal deposits across treatment phases.

**Supplementary Table 1**. Inter-rater agreement for specific OCT features.

| **OCT Feature** | **Cohen’s kappa** | **95% CI** | **Interpretation*** |
| --- | --- | --- | --- |
| Three-compartment infiltration classification |  |  |  |
| Sub-RPE deposits (present/absent) | 0.91 | 0.78–1.00 | Excellent |
| Subretinal deposits (present/absent) | 0.88 | 0.73–1.00 | Excellent |
| Intraretinal deposits (present/absent) | 0.86 | 0.67–1.00 | Excellent |
| Sub-RPE deposit subtypes |  |  |  |
| Thickened RPE (focal) vs. (diffuse) | 0.82 | 0.64–1.00 | Excellent |
| Shallow PED vs. large PED | 0.85 | 0.68–1.00 | Excellent |
| Subretinal deposit subtypes |  |  |  |
| Focal round | 0.80 | 0.60–1.00 | Good |
| Band-like | 0.83 | 0.65–1.00 | Excellent |
| Band-like with vitelliform lesions | 0.78 | 0.55–1.00 | Good |
| Intraretinal deposit subtypes |  |  |  |
| Incomplete VHRL | 0.84 | 0.62–1.00 | Excellent |
| Complete VHRL | 0.87 | 0.68–1.00 | Excellent |
| Other OCT features |  |  |  |
| EZ disruption | 0.90 | 0.77–1.00 | Excellent |
| EZ-RPE attenuation | 0.79 | 0.58–1.00 | Good |
| Preretinal deposits | 0.82 | 0.64–1.00 | Excellent |
| Vitreous cells | 0.88 | 0.73–1.00 | Excellent |

* Interpretation: κ < 0.20, poor; 0.21–0.40, fair; 0.41–0.60, moderate; 0.61–0.80, good; 0.81–1.00, excellent. CI, confidence interval; EZ, ellipsoid zone; OCT, optical coherence tomography; PED, pigment epithelial detachment; RPE, retinal pigment epithelium; VHRL, vertical hyperreflective lesion.

**Supplementary Table 2**. Sensitivity analysis of visual prognostic analysis restricted to eyes with direct intraocular biopsy confirmation.

|  | **Individual analysis** | | | | **Multivariable analysis** | | |
| --- | --- | --- | --- | --- | --- | --- | --- |
| **Feature** | **β** | **95% CI** | **P value** | **Q value (FDR)** | **β** | **95% CI** | **P value** |
| Baseline BCVA (logMAR) | 0.865 | 0.626 to 1.103 | < 0.001* | 0.001† | 0.584 | 0.145 to 1.023 | 0.018* |
| EZ-RPE attenuation^a^ | 1.346 | 0.749 to 1.942 | < 0.001* | 0.001† |  |  |  |
| EZ disruption | 1.201 | 0.706 to 1.696 | < 0.001* | < 0.001† | 0.529 | 0.043 to 1.016 | 0.036* |
| Intraretinal deposits | 0.89 | 0.14 to 1.641 | 0.024* | 0.043† | 0.044 | -0.502 to 0.59 | 0.859 |
| Sub-RPE deposits | 0.719 | -1.41 to 2.849 | 0.188 | 0.242 |  |  |  |
| Subretinal deposits | 0.82 | 0.278 to 1.362 | 0.007* | 0.016† | 0.167 | -0.124 to 0.458 | 0.229 |
| Vitreous cells | 0.372 | -0.385 to 1.129 | 0.309 | 0.348 |  |  |  |
| Preretinal deposits | -0.098 | -0.797 to 0.601 | 0.768 | 0.768 |  |  |  |
| CNS involvement | -0.449 | -1.113 to 0.216 | 0.160 | 0.240 |  |  |  |

*P < 0.05. †Q < 0.05 (Benjamini–Hochberg false discovery rate correction). FDR, false discovery rate; BCVA, best-corrected visual acuity; logMAR, logarithm of the minimum angle of resolution; CI, confidence interval; CNS, central nervous system; EZ, ellipsoid zone; RPE, retinal pigment epithelium.

^a^EZ-RPE attenuation was excluded from the multivariable model owing to its conceptual and statistical overlap with EZ disruption (Phi coefficient = 0.690; VIF = 2.32).

**Supplementary Figure 1**. OCT features of sub-RPE and subretinal deposits across treatment phases.


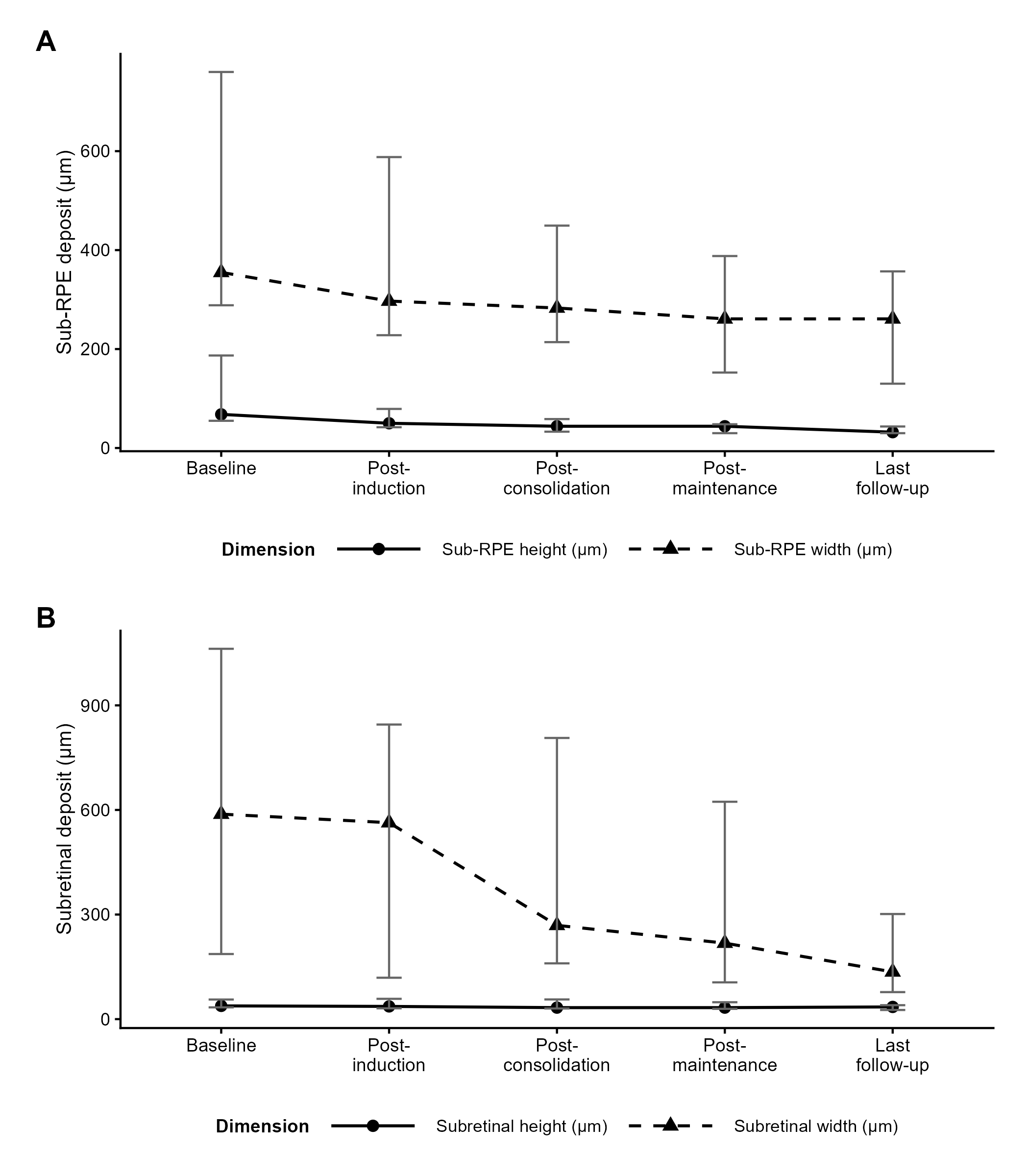

Supplement: Supplementary file 1 [file Table_1.DOCX]
